# The effect of variations in CT scan protocol on femoral finite element failure load assessment using phantomless calibration

**DOI:** 10.1371/journal.pone.0265524

**Published:** 2022-03-18

**Authors:** Ali Ataei, Jelle Eikhout, Ruud G. H. van Leeuwen, Esther Tanck, Florieke Eggermont

**Affiliations:** 1 Orthopaedic Research Lab, Radboud Institute for Health Sciences, Radboud University Medical Center, Nijmegen, The Netherlands; 2 Department of Radiotherapy, Radboud Institute for Health Sciences, Radboud University Medical Center, Nijmegen, The Netherlands; New York University Langone Health, UNITED STATES

## Abstract

Recently, it was shown that fracture risk assessment in patients with femoral bone metastases using Finite Element (FE) modeling can be performed using a calibration phantom or air-fat-muscle calibration and that non-patient-specific calibration was less favorable. The purpose of this study was to investigate if phantomless calibration can be used instead of phantom calibration when different CT protocols are used. Differences in effect of CT protocols on Hounsfield units (HU), calculated bone mineral density (BMD) and FE failure loads between phantom and two methods of phantomless calibrations were studied. Five human cadaver lower limbs were scanned atop a calibration phantom according to a standard scanning protocol and seven additional commonly deviating protocols including current, peak kilovoltage (kVp), slice thickness, rotation time, field of view, reconstruction kernel, and reconstruction algorithm. The HUs of the scans were calibrated to BMD (in mg/cm^3^) using the calibration phantom as well as using air-fat-muscle and non-patient-specific calibration, resulting in three models for each scan. FE models were created, and failure loads were calculated by simulating an axial load on the femur. HU, calculated BMD and failure load of all protocols were compared between the three calibration methods. The different protocols showed little variation in HU, BMD and failure load. However, compared to phantom calibration, changing the kVp resulted in a relatively large decrease of approximately 10% in mean HU and BMD of the trabecular and cortical region of interest (ROI), resulting in a 13.8% and 13.4% lower failure load when air-fat-muscle and non-patient-specific calibrations were used, respectively. In conclusion, while we observed significant correlations between air-fat-muscle calibration and phantom calibration as well as between non-patient-specific calibration and phantom calibration, our sample size was too small to prove that either of these calibration approaches was superior. Further studies are necessary to test whether air-fat-muscle or non-patient-specific calibration could replace phantom calibration in case of different scanning protocols.

## Introduction

Bone metastases occur in a large number of breast, prostate, thyroid, lung and kidney cancer patients [1]. These metastases can be painful and can increase the risk of pathological fractures. Such fractures cause mobility problems resulting in the inability to perform activities of daily living, leading to a reduced quality of life and an increased mortality risk [2].

It is of great importance to accurately determine the fracture risk of these patients, so the appropriate treatment can be chosen. In current clinical practice, assessment of fracture risk is done using computed tomography (CT) scans or conventional radiography, however the assessment of the risk has been shown to be quite complex [3]. Mirels’ scoring system is a commonly used method which calculates fracture risk based on the site and location of the lesion, as well as the appearance and severity of pain [4,5]. However, several studies showed that the Mirels’ score lacked specificity [3,6–8]. Van der Linden et al. introduced a 30 mm axial cortical involvement as a simple criterion assisting clinicians to select the appropriate treatment [6], which was recently validated [7]. Using axial cortical involvement, specificity increased compared to Mirels’ score whereas sensitivity was comparable [9,10]. Since such assessments are mainly relying on the clinician’s ability to interpret a radiographic image, patients might be either overtreated, involving unnecessary and cost-ineffective interventions, or might be undertreated, causing fractures and decreased quality of life because of further complications [3,11].

Patient-specific finite element (FE) modeling can predict fracture risk with higher accuracy compared to the methods currently used by clinicians [11–14]. Input to the FE models can be CT scans [11,13,14] as well as MR images [15]. CT scans need to be calibrated to convert the Hounsfield units (HU) to bone mineral density (BMD) to calculate the mechanical properties of the bone. For this purpose, usually a calibration phantom [13,14,16–18] is scanned along with the patient. However, these phantoms are expensive and impractical. Furthermore, since they have to be scanned together with the patient, it is not possible to generate FE models of scans performed without a calibration phantom. In some studies, phantomless calibration methods are used to obtain patient-specific calibration functions, for example based on HU of intrinsic patient tissue such as fat, muscle, air or blood [19–24]. Such phantomless calibration methods appear to result in similar bone mineral densities compared to calibration with phantoms [16–22,25]. However, there is limited information about how these methods affect failure load assessment using FE modeling. A few previous studies showed that their proposed methods of phantomless calibration, by using internal tissues to calculate a calibration function specific for each patient [20,26] or by applying the same general calibration function for all patients, i.e. non-patient-specific calibration [27], was applicable to FE studies on retrospective cohorts lacking a calibration phantom [20,26,27].

Recently, Eggermont et al. developed a novel method that calibrates the CT scan based on the HU of air, fat, and muscle tissue and studied the influence of this air-fat-muscle calibration on femoral FE failure load assessment [19]. They found a very high correlation (R^2^ = 0.94) between phantom calibration and air-fat-muscle method, and accordingly, no significant difference in FE failure loads between the phantom calibration and the air-fat-muscle calibration. They also applied a non-patient-specific calibration. Although the non-patient-specific calibration was less favorable than the air-fat-muscle calibration, it also highly correlated to phantom calibration (R^2^ = 0.94) [19]. This suggests that air-fat-muscle and non-patient-specific calibration might be good alternatives for phantom calibration, which could allow fracture risk assessment based on FE modeling without the effort of scanning a calibration phantom along with the patient. Additionally, these methods have the potential to enable the use of phantomless CT scans retrospectively in order to validate the FE models developed for fracture risk assessment.

However, the study by Eggermont et al. only tested the air-fat-muscle and non-patient-specific calibration methods on well-protocolized CT scans [19]. It is known that changes in scan protocol can result in different HU, BMD and FE failure loads [28–34]. Depending on certain patient properties such as size, age or body part(s) that are to be scanned, deviations in scanning protocols are frequently made, e.g., to alter the radiation dose. It is known that deviations in scanning protocols can affect the HU, potentially resulting in different BMD and calculated failure loads [28–39]. For example, differences in reconstruction kernel can significantly influence BMD measurements and failure load calculated by FE models using phantom calibration [28,29,31].

For air-fat-muscle calibration and non-patient-specific calibration, the influence of differences in scan protocol has not been studied yet. Evaluation of the influence of commonly changed CT protocols on FE models when using phantomless calibration instead of phantom calibration is necessary to find out if these calibrations are equally feasible to use as phantom calibration. Therefore, the aim of this study is to investigate if air-fat-muscle calibration or non-patient-specific calibration can be used instead of phantom calibration when different CT protocols are used. Differences in effect of CT protocols on HU, BMD and FE failure loads between phantom and the two phantomless calibration methods were studied.

## Methods

### CT scanning

Five fresh frozen human lower limbs (3 female, 2 male; mean age 70.4, range 63–77; 2 left, 3 right) were obtained from the Anatomy Department of the Radboud university medical center (Radboudumc, https://www.radboudumc.nl/en/research/departments/anatomy) according to the Dutch Body Donation Program for Science and Education [40].

All lower limbs were scanned on a Philips Brilliance Big Bore (Philips Medical Systems, Eindhoven, The Netherlands) in supine human orientation (patella facing up, proximal end first) on top of a solid calibration phantom (Image analysis, Columbia, KY). This calibration phantom contained four rods with known calciumhydroxyapatite (CaHA) concentrations (0, 50, 100, and 200 mg/cm^3^) and can be used to calibrate the HU of the scan to CaHA density as a measure of BMD (in mg/cm^3^) [11,12,19,29,41–43]. All limbs were scanned using the standard protocol that we use for FE modeling [11,12,19] and with seven additional commonly used protocols with variations on this standard protocol which were selected based on the literature [31,34,35,37–39,44], as well as our observance in our previous patient studies [12,29] (Table 1). Eight different scans per leg and 40 scans in total were made. For all protocols, the pitch was set at 0.813.

**Table 1:** Overview of the different scan protocols and the corresponding CT settings. Standard protocol covers the CT settings that are standard for scans made at the Radiotherapy department of the Radboudumc. All other protocols deviate one setting from the standard protocol.

	Value in standard protocol	Value in deviating protocol
**Current (mAs)**	**220**	163
**kVp**	**120**	140*
**Slice thickness (mm)**	**3**	1
**Rotation time (s)**	**1**	0.75
**FOV (mm)**	**480**	600
**Reconstruction kernel**	**B (for bone imaging)**	C (for sharper images)
**Reconstruction algorithm**	**standard**	iDose4

*For the 140 kVp scan, the scanner software did not allow the standard tube current of 220 mAs. Hence, we used the highest value possible, which was 218 mAs.

### FE modeling

**Fig 1:**
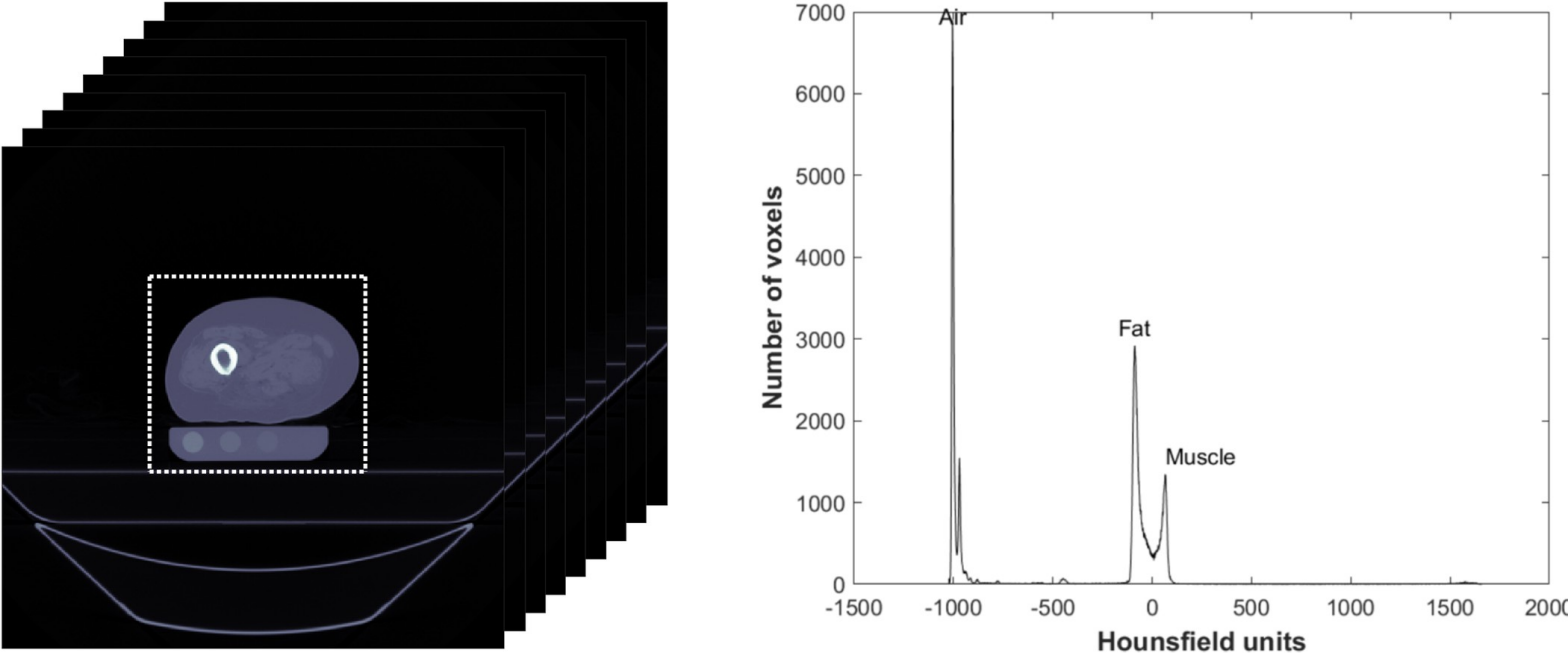
An example of the selected region of interest (left) and the histogram of the Hounsfield units within the region of interest with the peaks for air, fat, and muscle (right).

Subject-specific FE models were created for each femur based on the CT scan made with the standard protocol, as described before [11,12,42]. Subject-specific femoral geometry was obtained from the CT scan made with the standard protocol by selecting the voxels containing femoral tissue (Mimics 14.0, Materialise, Leuven, Belgium). Femoral geometries were modeled to a solid mesh consisting of tetrahedral elements (Patran 2011, MSC Software Corporation, Santa Ana, CA, USA). The femoral geometry was registered to all other CT scans (elastix [45,46]), followed by an accuracy check. The HU of each element was calibrated to BMD (in mg/cm^3^) using the air-fat-muscle, non-patient-specific, and phantom calibration [19]. All the mentions of BMD in the study indicate “calculated BMD”. For phantom calibration, nine diaphyseal slices were selected. The HU within the rods of the phantom in these slices were linearly correlated to the known CaHA concentrations to obtain the phantom calibration function. For air-fat-muscle calibration, a square region of interest was defined around the leg, including approximately 1 cm of air on each side of the leg, on the same nine diaphyseal slices that were used for the phantom calibration (Fig 1 left). The peaks of air, fat, and muscle tissue were extracted from a combined histogram of all HUs in the region of interest (Fig 1 right). Subsequently, to obtain the air-fat-muscle calibration function, the extracted HU peaks were linearly fitted to the reference BMD values (-838 mg/cm^3^ for air, -86 mg/cm^3^ for fat and 35 mg/cm^3^ for muscle respectively) for each subject [19]. For the non-patient-specific calibration we used the same function as determined previously (BMD = 0.82 x HU−4.2 [19]), which was determined by averaging calibration functions of 26 patients. This calibration function was then applied to each of the 5 subjects.

**Fig 2:**
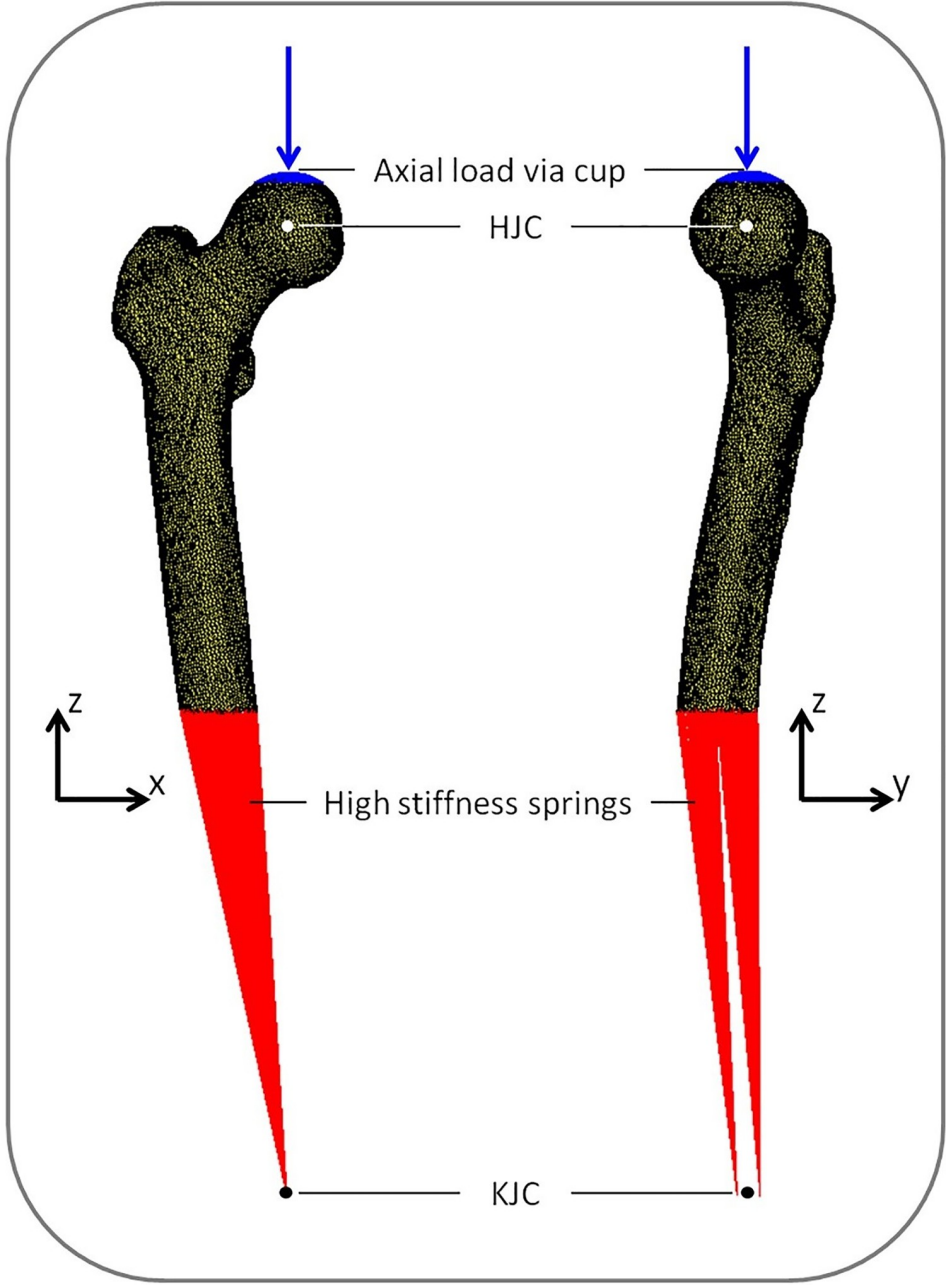
Boundary conditions of the finite element model. The model was distally fixated by two high stiffness springs, and the load was applied through a cup located on the head of the femur. The figure is with permission adapted from Eggermont et al., Bone, 2020 [11].

The BMD values (in mg/cm^3^) were eventually converted to ash densities (ρ_ash_ = 0.000887 x BMD + 0.0633) which were converted to non-linear isotropic material properties [47]. Some material properties were different between trabecular and cortical bone (threshold BMD = 250 mg/ cm^3^), using elastic modulus (MPa) = 14900 x ρ_ash_^1.86^; ultimate stress (MPa) = 102 x ρ_ash_^1.80^; plastic strain (mm/mm) at initial plastic phase = 0.00189 + 0.0241 x ρ_ash_ for BMD ≤ 250 mg/cm^3^ and 0.0184 − 0.0100 x ρ_ash_ for BMD > 250 mg/cm^3^; plastic modulus (MPa) of strain softening phase = −2080 x ρ_ash_^1.45^ for BMD ≤ 250 mg/cm^3^ and − 1000 for BMD > 250 mg/cm^3^; and stress (MPa) at the indefinite perfectly plastic phase = 43.1 x ρ_ash_^1.81^. The model was distally fixated by two bundles of high-stiffness springs. A cup representing the acetabulum was simulated on top of the femoral head and applied a displacement-driven load in the axial direction with increments of 0.1 mm (Fig 2). FE simulations were carried out using MSC.MARC (v2013.1, MSC Software Corporation, Santa Ana, CA, USA). Force-displacement (FD) curves were generated based on the displacement of the cup and the contact forces in the axial direction. It was assumed that a fracture occurred when the maximum total reaction force at the interface of the cup and femoral head was reached in the non-linear part of the load-displacement curve, which was defined as the failure load. For every femur, only BMD and subsequent mechanical properties varied between protocols, whereas all other model properties were left unchanged.

### Outcome measures

**Fig 3:**
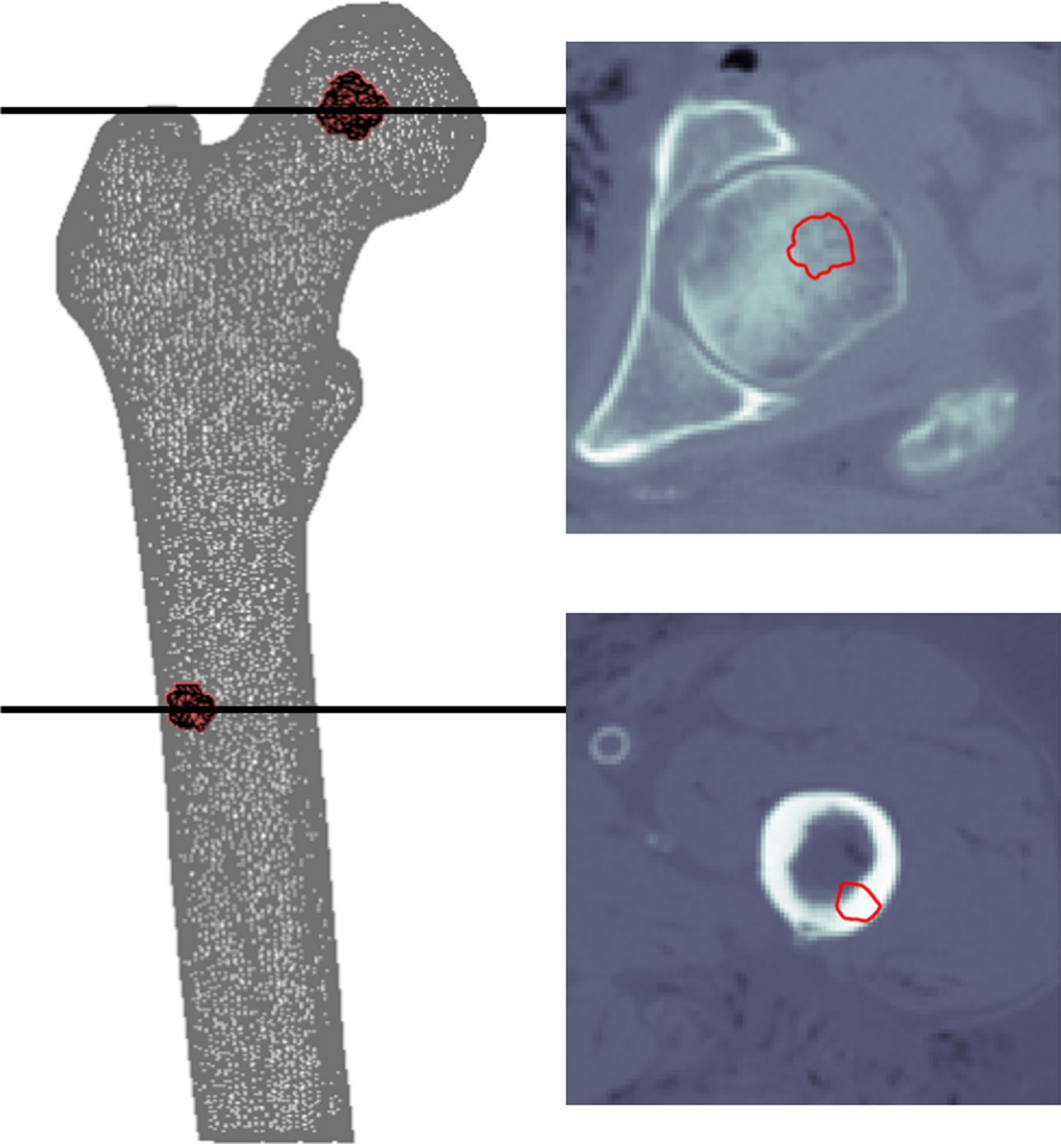
An example of the trabecular (upper) and cortical (lower) regions of interest including axial views.

To analyze HU and BMD, a cortical and a trabecular region of interest (ROI) were selected by selecting elements along the cortex of the femoral shaft and center of the femoral head, respectively (Fig 3). For each ROI, HUs were obtained, which were subsequently calibrated to BMDs using the phantom calibration [11,12,19,29], air-fat-muscle calibration [19], and non-patient-specific calibration [19].

Additionally, we calculated FE failure loads based on phantom, air-fat-muscle, and non-patient-specific calibration. For every protocol and calibration method, the HU and BMD within the cortical and trabecular ROIs, as well as the failure loads, were analyzed and compared. Basic statistics, i.e. averages and correlations were calculated.

## Results

### Hounsfield unit

**Fig 4:**
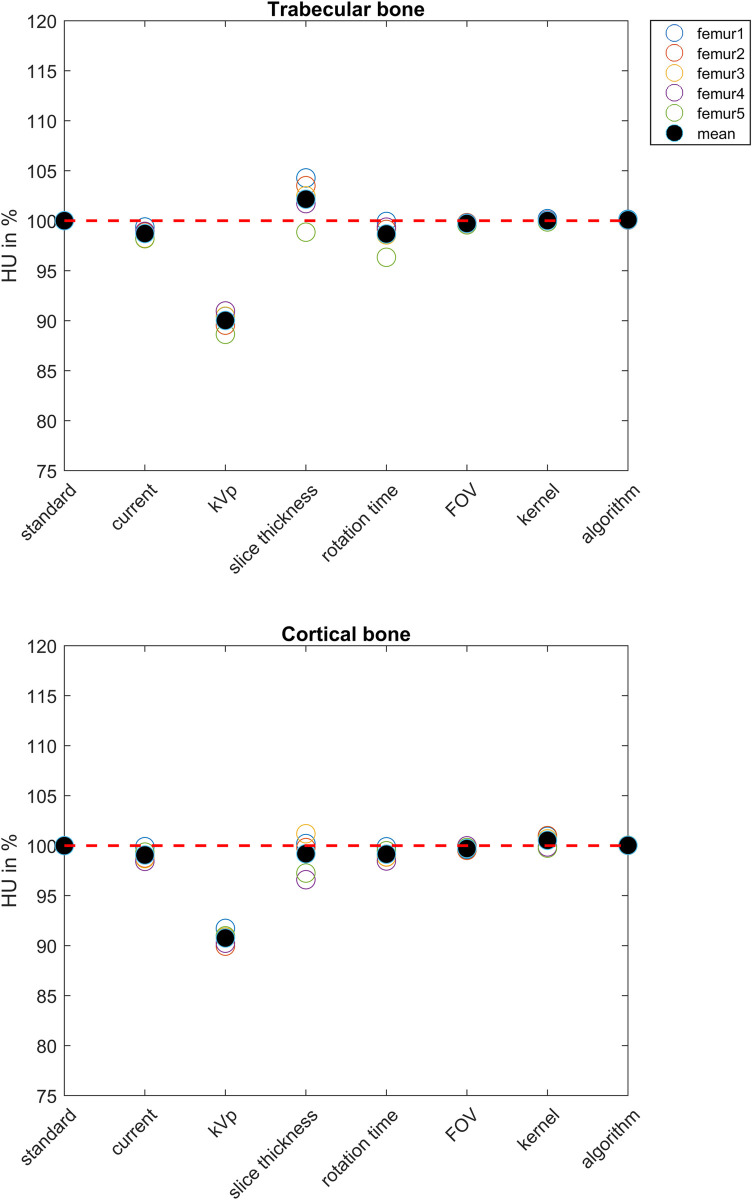
HU of the trabecular (upper figure) and cortical (lower figure) ROIs. For each femur, the HUs are displayed relative to the standard protocol. The red dashed line indicates 100%.

Mean HU in trabecular and cortical ROI decreased with 10% and 9.2% when changing the kVp from 120 to 140, respectively. All other protocol variations had little effect, i.e. on average less than 2%, on the HU (Fig 4).

### Bone mineral density

**Fig 5:**
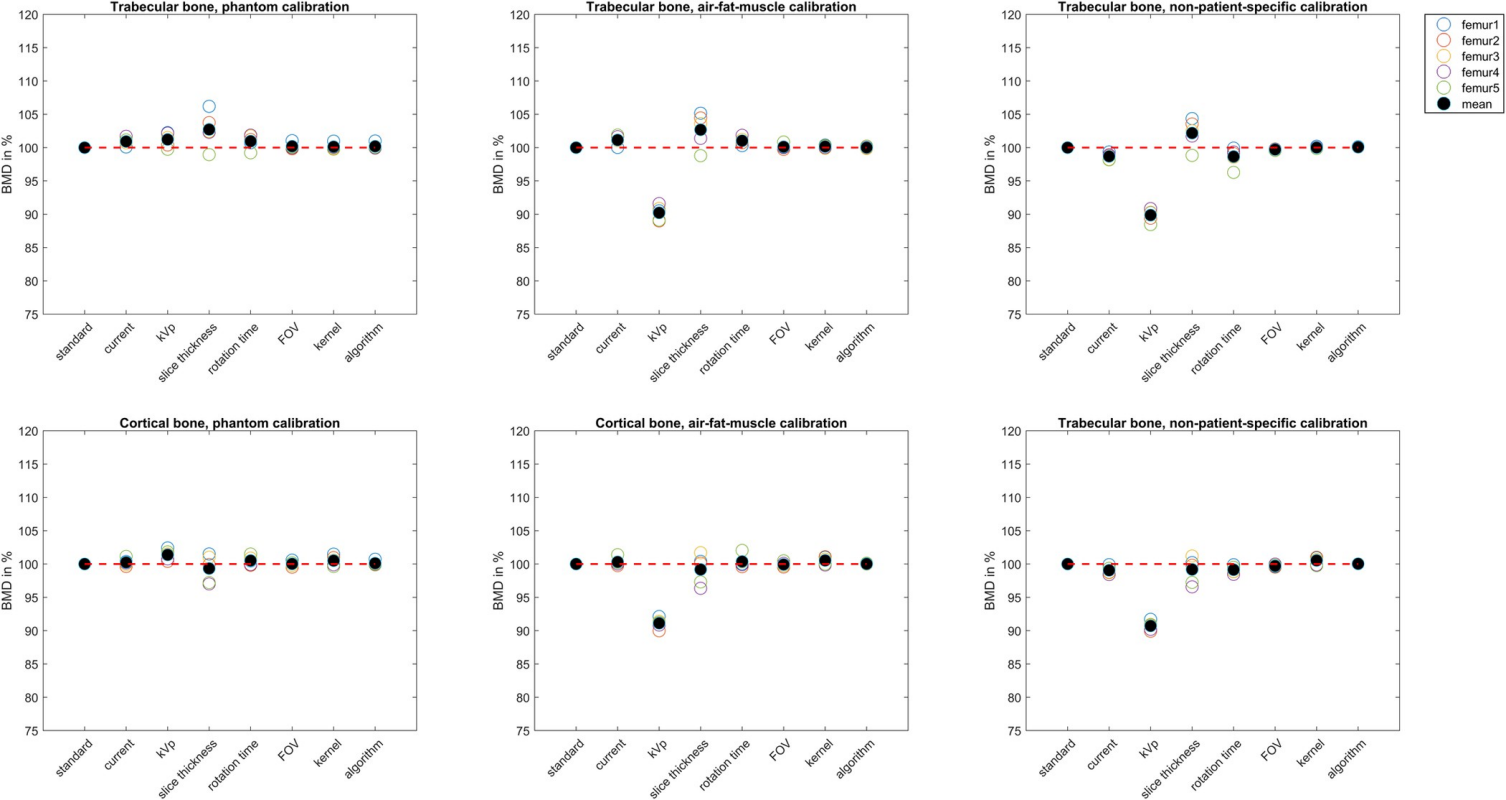
BMD of the trabecular (upper half) and cortical bone (lower half) for phantom (left), air-fat-muscle (middle), and non-patient-specific (right) calibration per protocol. For each femur, the BMD values are displayed relative to the standard protocol. The red dashed line indicates 100%.

Generally, BMDs were 3.3% and 3.9% higher after air-fat-muscle and non-patient-specific calibrations compared to phantom calibration, respectively. Considering the standard protocol, the average absolute BMDs of cortical bone were 867 mg/cm^3^ (range 666 to 1084 mg/cm^3^) for phantom calibration, 895 mg/cm^3^ (range 743 to 1089 mg/cm^3^) for air-fat-muscle calibration, and 912 mg/cm^3^ (range 701 to 1126 mg/cm^3^) for non-patient-specific calibration. For trabecular bone, these values were 246 mg/cm^3^ (range 214 to 269 mg/cm^3^), 254 mg/cm^3^ (range 208 to 281 mg/cm^3^) and 267 mg/cm^3^ (range 231 to 296 mg/cm^3^), respectively. When phantom calibration was used, the relative BMD showed very little variation between the different protocols (Fig 5). For both air-fat-muscle and non-patient-specifc calibration, the BMD after changing kVp from 120 to 140 was relatively lower: for trabecular bone, the differences with the standard protocol were 9.8% and 10.1%, respectively, and for cortical bone, the differences with the standard protocol were 8.9% and 9.3% respectively (Fig 5).

### Failure load

**Fig 6:**
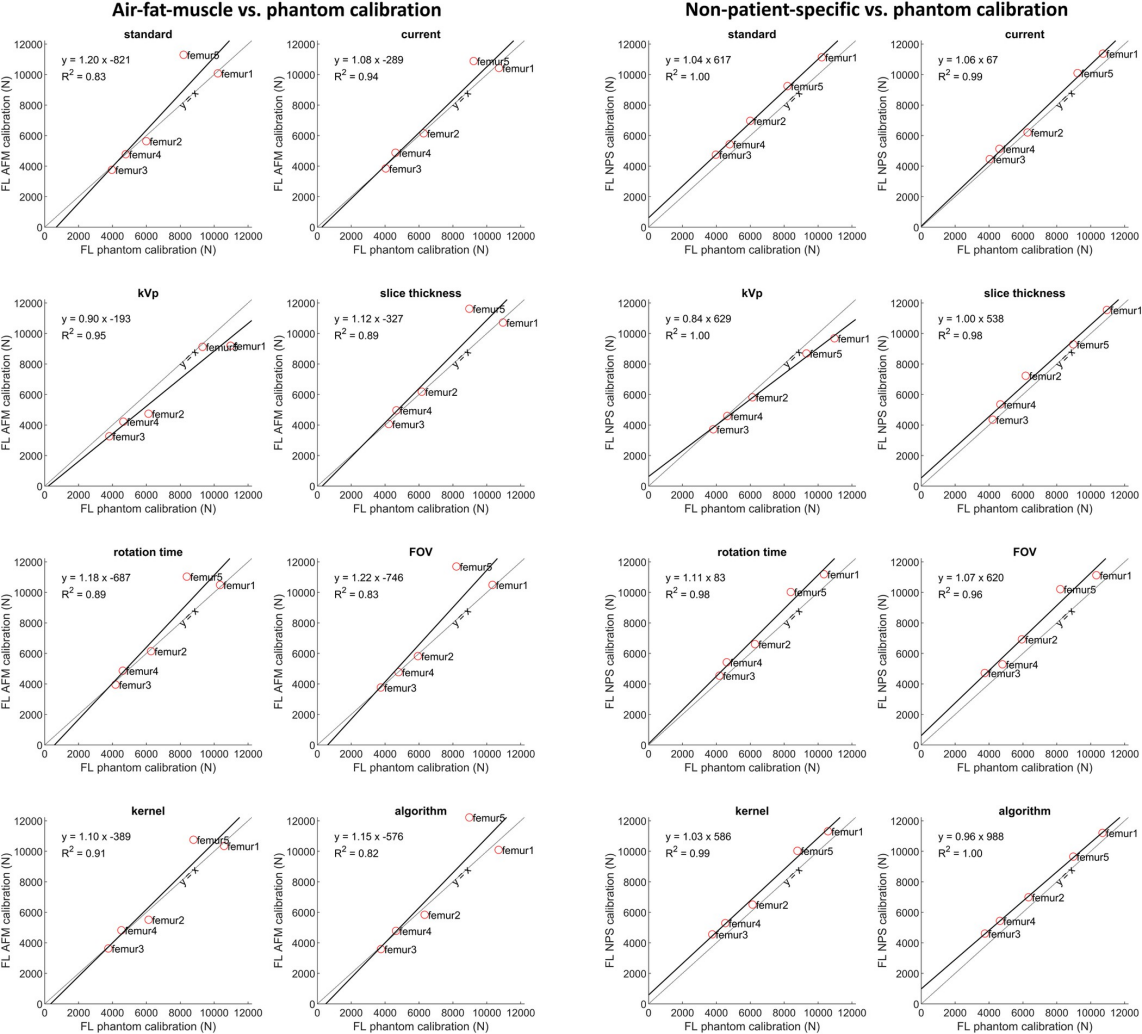
Correlation between failure loads after phantom and air-fat-muscle calibration and phantom and non-patient-specific calibration. Solid lines show the best-fit lines for each protocol.

The failure load of the separate femurs obtained from FE analysis showed strong correlation between calibration methods (Fig 6). Only femur 5 showed a remarkable overestimation in failure load after air-fat-muscle calibration compared to phantom calibration. Higher correlations between phantom and air-fat-muscle calibration were obtained when excluding femur 5 (S1 Fig).

**Fig 7:**
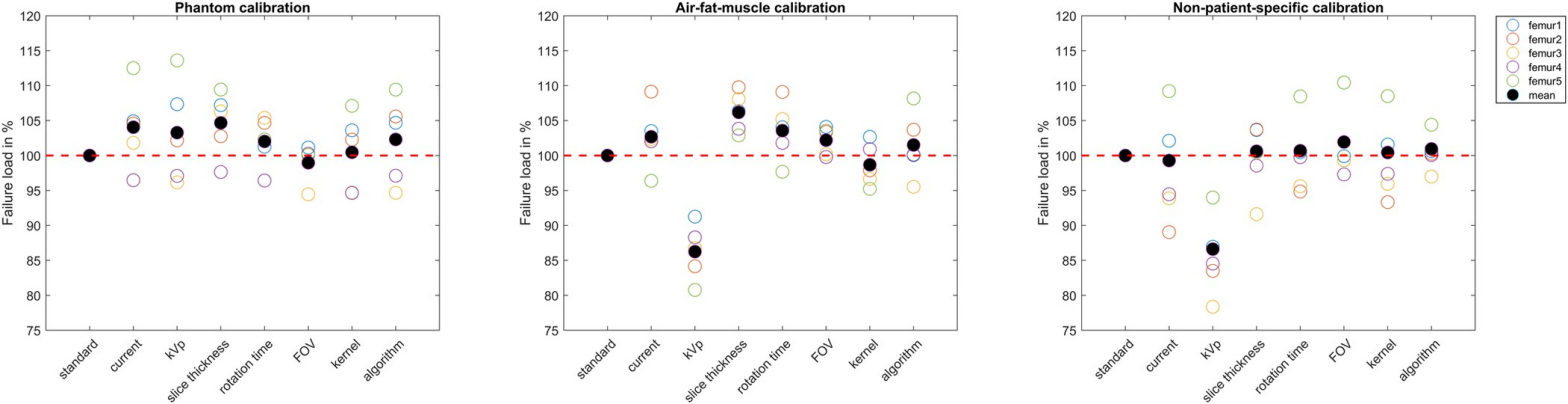
Failure load per protocol, per calibration method. For each femur, the failure loads are displayed relative to the standard protocol. The red dashed line indicates 100%.

The average failure load for the standard protocol was 6632 N (range 3967 to 10218 N) after phantom calibration, 7110 N (range 3759 to 11297 N) after air-fat-muscle calibration and 7505N (range 4749N to 11136 N) after non-patient-specific calibration. The difference in failure load relative to the standard protocol varied between femurs (Fig 7). Maximum mean failure load variations from the standard protocol were 4.7% for phantom calibration, 13.8% for air-fat-muscle calibration, and 13.4% for non-patient-specific calibration. Most noticable were the relatively large decreases in failure load with 13.8% and 13.4% when changing kVp from 120 to 140 after air-fat-muscle and non-patient-specific calibration, respectively.

## Discussion

The aim of this study was to investigate the differences in effect of common variations in CT scan protocols on HU, BMD and FE failure loads between phantom and air-fat-muscle calibration and phantom and non-patient-specific calibration, to determine whether air-fat-muscle and non-patient-specific calibration perform similar to phantom calibration. Most of the protocols had little effect on HU. Only the mean HU in trabecular and cortical ROI decreased when changing the kVp from 120 to 140. Absolute BMDs were higher after air-fat-muscle calibration compared to phantom calibration and were even higher after non-patient-specific calibration. The relative BMD showed very little variation for phantom calibration. The difference in failure load between the standard protocols for phantom and air-fat-muscle as well as non-patient-specific calibration was very small and both phantomless methods showed a strong correlation with phantom calibration. Per femur, the effect of the deviating protocols on the failure load varied considerably. On average, changing kVp from 120 to 140 resulted in a relatively large decrease in failure load for the air-fat-muscle as well as the non-patient-specific calibration methods in comparison to the phantom calibration. The other variations did not noticeably deviate in failure load in comparison to the standard protocol.

The strong correlation between phantom and air-fat-muscle calibration methods allows their use to be interchangeable in case the air, fat, and muscle peaks are clearly detectable in the HU histogram. Also phantom and non-patient-specific calibration were strongly correlated. Hence non-patient-specific calibration is also useable, although in a previous study including 67 femurs using the standard protocol, a small and significant difference in failure load between phantom and non-patient-specific calibration was present [19].

One must be critical about the implications that minor differences can have on the eventual failure risk assessment in patients. The treatment that a patient receives is partially based on a certain fracture risk threshold: a risk below the threshold requires conservative radiotherapy and a risk above the threshold requires stabilizing surgery [11]. When the fracture risk is close to the threshold, a minor failure load difference between calibration methods can influence the treatment plan. However, besides the calculated fracture risk, clinicians take other patient factors such as pain level, clinical condition, and life expectancy into consideration as well to come to a thoughtful treatment plan. As for the fracture risk assessment based on retrospective CT scans and comparing this with actual fracture occurrence, air-fat-muscle as well as non-patient-specific calibration seem to be a reliable alternatives, not only for metastatic femurs, but probably also for CT scans of patients with osteoporosis.

**Fig 8:**
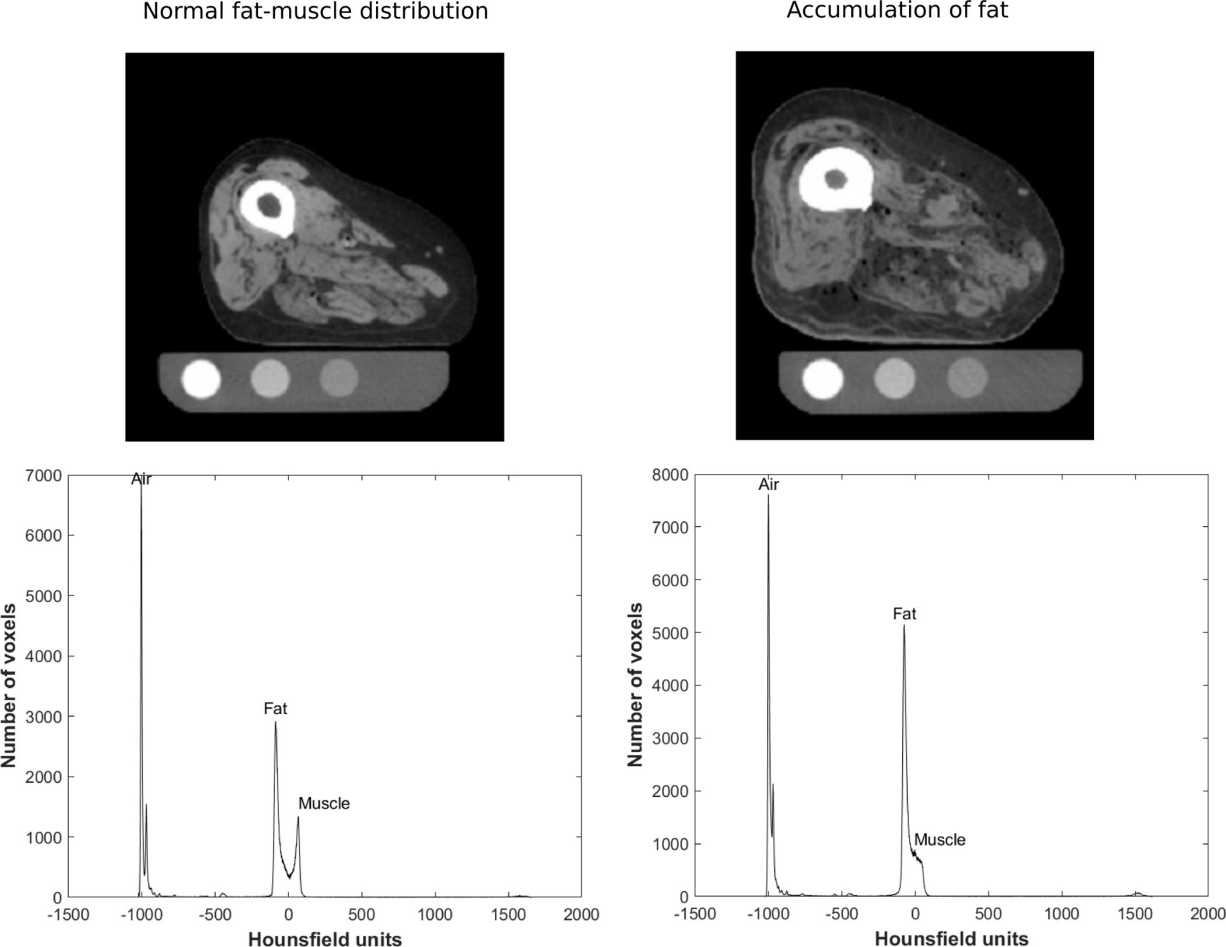
Slices of the same diaphyseal region from the CT scans of femur 4 (left) and femur 5 (right) with the corresponding HU histograms. Femur 5 shows accumulation of fat in the muscle tissue.

An exception to the strong correlation between the phantom and air-fat-muscle calibration methods was femur 5, which showed a much higher failure load after air-fat-muscle calibration (Fig 8). Upon inspection of the scans of this femur, accumulation of fat in muscle tissue was observed, causing a downgrade of muscle quality and density (Fig 8). Therefore, one should check whether the fat and muscle peaks are clearly detectable prior to using air-fat-muscle calibration.

The influence of the different scan protocols on the failure load with respect to the standard protocol varied per femur. Maximum mean failure load differences were 4.7% for phantom calibration, 13.8% for air-fat-muscle calibration and 13.4% for non-patient-specific calibration, implying that phantom calibration after scanning with deviating protocols returns more robust results than air-fat-muscle or non-patien-specific calibration.

Changing kVp showed the highest relative difference in failure load after air-fat-muscle and non-patient-specific calibration compared to the standard protocol. Giambini et al. investigated the influence of varying kVp values on the HU of bone [31]. They showed that increasing kVp caused a drop in HU. On the other hand, they found little variation in cortical BMD between the two kVp values. These findings concur with the results of the phantom calibrated scans found in the present study: the HU of the calibration phantom rods were relatively lower for the kVp protocol compared to the standard protocol (S1 Table), but the BMD barely differed between protocols. Subsequently, no remarkable differences in failure load were observed. Apparently, in contrast with the air-fat-muscle and non-patient-specific calibration, the phantom calibration method is able to correct for any changes in kVp, thus nullifying the influence of the decreased HU on the calibration. For the air-fat-muscle calibration, this can be explained by the fact that HU are less influenced by kVp differences when the electron density of the tissues gets below the electron density of water [48], and thus the effect of kVp deviations might be limited in fat and muscle tissue [49]. Hence, the calibration curve of air-fat-muscle is practically similar to the standard protocol, even though the HU of bone will be lower because of the higher kVp. On the contrary, as the densities of the rods in the calibration phantom are higher than water, the effect of kVp deviations is more noticeable, resulting in a different calibration curve that is able to correct for the change in kVp in bone HU. Additionally, it is evident that non-patient-specific calibration cannot correct for any differences caused by changes in kVp. Hence, it is advised to use the same kVp for the scans that are calibrated with air-fat-muscle or non-patient-specific methods.

Additionally, one should notice that reconstruction methods differ between different types of CT scanners. Following a standard protocol for one scanner might therefore not yield the same BMD on another scanner, potentially resulting in a different failure risk calculation [28,32]. Eggermont et al. found significant differences in HU (average 7±2%) and BMD (max 6±1%) between four different scanners using phantom calibration, whereas failure load did only significantly differ in one scan as opposed to the other three (max 17±5%) [29]. Birnbaum et al. support this finding and observed a significant 9–10 HU difference between scanners in renal cyst tissue [36]. Non-patient-specific calibration will not be able to correct for any differences between CT scanners. Future research should point out how different scanners affect air-fat-muscle calibration and resulting failure load.

Additionally, in our FE simulation only one axial loading condition was applied. However, our FE model with the relatively simple axial loading condition showed an improvement of fracture risk assessments compared to methods used in clinical guidelines in several previous studies [11,12]. Although multiple loading conditions may lead to more information about the fracture risk, using one loading condition was very useful for clinical implementation of the FE model [50].

We acknowledge some limitations in this study. First of all, a relatively small number of cadaveric legs was used in the present study. Due to the small sample size we were not able to conduct any reliable statistical analysis. However, the correlation between failure load after phantom calibration and failure load after air-fat-muscle calibration was comparable with the data from a study by Eggermont et al. (S2 Fig) in which 67 femurs were included [19]. Therefore, we think it is plausible that kVp significantly affects the HU, BMD, and failure load compared to the other protocol variations. Remarkably, the femurs calibrated with non-patient-specific calibration seemed to deviate more from the data of Eggermont et al. [19]. This might be explained by the fact that the femurs in the current study were scanned as single legs, whereas the femurs of Eggermont et al. were actual patients. When scanning a single leg, the HU will be higher because of less absorption of other tissue (due to a missing contralateral leg). As a result the calculated failure loads will be too high, for which non-patient-specific calibration apparently cannot correct, whereas air-fat-muscle calibration seems to be able to do this better. A second limitation relates to the effect of cryopreservation on subjects. It is known that freezing and subsequent thawing can influence the HU of tissue because of differences in water content [51,52], which might have affected the subjects in this study. However, as we compare three calibration methods and use the same material for all methods, the effect of freezing and thawing on the conclusions of this study are expected to be negligible. Moreover, conducting a similar study with living subjects would be unethical due to the high radiation doses. A third limitation was the lack of data on how the subjects passed away and if any diseases played a role. These factors could have had an influence on the tissues of the subjects, and subsequently on the BMD and failure load after air-fat-muscle calibration. Femur 5 is such an example. Finally, we only tested a few protocol variations. Since we could not investigate the effect of all possible variations in CT scan protocols and we are aiming at increasing the clinical implementation of femoral fracture risk assessment, we selected the protocols based on the literature, as well as protocol violations that we came across doing our clinical patient study. These protocol variations were subsequently examined in this study. We only tested the protocols on one CT scanner. In the future, it should be investigated whether the air-fat-muscle calibration varies compared to phantom calibration when using different CT scanners.

In conclusion, while we observed significant correlations between air-fat-muscle calibration and phantom calibration as well as between non-patient-specific calibration and phantom calibration, our sample size was too small to prove that either of these calibration approaches was superior. In addition, our approaches for air-fat-muscle and non-patient-specific calibration should not be used for variations in kVp. These should be corrected for. The influence of tissue abnormalities on air-fat-muscle calibration compared to phantom calibration should be studied explicitly to prevent discrepancies in fracture risk assessment. Further studies are necessary to test whether air-fat-muscle or non-patient-specific calibration could replace phantom calibration in case of different scanning protocols and different CT scanners.

## Supporting information

S1 Fig Correlation between failure loads after phantom and air-fat-muscle calibration. Solid lines show the best-fit lines for each protocol in which femur 5 was excluded due to the accumulation of fat.
(TIF)

S2 Fig Correlation between the failure load of the femurs of the previous study (Eggermont et al., PLOS ONE, 2019; blue line, n = 67) compared to the correlation of the current study, left for air-fat-muscle and right for non-patient-specific calibration. This figure shows that in case of air-fat-muscle calibration four of the five femurs are within the range of the patients studied by Eggermont et al. (red line, n = 4). Only the femur with the deviating fat and muscle balance is deviating. Additionally, it can be seen that the correlations between phantom and air-fat-muscle calibration are comparable when excluding the deviating femur. The femurs calibrated with non-patient-specific calibration seem to deviate more from the femurs of the previous study. This might be explained by the fact that the femurs in the current study are scanned as single legs, whereas the femurs of the previous study were actual patients. When scanning a single leg, the HU will be higher because of less absorption of other tissue (due to a missing contralateral leg). As a result, the failure loads will be too high, for which non-patient-specific calibration apparently cannot correct, whereas air-fat-muscle calibration seems to be able to do this better.
(TIF)

S1 Table Mean HUs per protocol for each calibration phantom rod. (DOCX)

S1 Data (ZIP)

## References

[1] Johnson SK, Knobf MT. Surgical interventions for cancer patients with impending or actual pathologic fractures. Orthopaedic Nursing. 2008;27(3):160–71. doi: 10.1097/01.NOR.0000320543.90115.d5 18521030

[2] Ottanelli S. Prevention and treatment of bone fragility in cancer patient. Clin Cases Miner Bone Metab. 2015;12(2):116–29. doi: 10.11138/ccmbm/2015.12.2.116 26604936 PMC4625767

[3] Van der Linden YM, Dijkstra PD, Kroon HM, Lok JJ, Noordijk EM, Leer JW, et al. Comparative analysis of risk factors for pathological fracture with femoral metastases. J Bone Joint Surg Br. 2004;86(4):566–73. 15174555

[4] Ulaner GA, Zindman AM, Zheng J, Kim TW, Healey JH. FDG PET/CT Assesses the Risk of Femoral Pathological Fractures in Patients With Metastatic Breast Cancer. Clin Nucl Med. 2017;42(4):264–70. doi: 10.1097/RLU.0000000000001580 28166159 PMC5334437

[5] Mirels H. Metastatic disease in long bones. A proposed scoring system for diagnosing impending pathologic fractures. Clin Orthop Relat Res. 1989(249):256–64. 2684463

[6] Damron TA, Morgan H, Prakash D, Grant W, Aronowitz J, Heiner J. Critical evaluation of Mirels’ rating system for impending pathologic fractures. Clinical Orthopaedics and Related Research®. 2003;415:S201–S7. doi: 10.1097/01.blo.0000093842.72468.73 14600611

[7] Tatar Z, Soubrier M, Dillies AF, Verrelle P, Boisgard S, Lapeyre M. Assessment of the risk factors for impending fractures following radiotherapy for long bone metastases using CT scan-based virtual simulation: a retrospective study. Radiation oncology. 2014;9(1):227. doi: 10.1186/s13014-014-0227-1 25319635 PMC4205287

[8] Shimoyama T, Katagiri H, Harada H, Murata H, Wasa J, Hosaka S, et al. Fracture after radiation therapy for femoral metastasis: incidence, timing and clinical features. Journal of Radiation Research. 2017;58(5):661–8. doi: 10.1093/jrr/rrx038 28992299 PMC5737329

[9] van der Linden YM, Kroon HM, Dijkstra SP, Lok JJ, Noordijk EM, Leer JWH, et al. Simple radiographic parameter predicts fracturing in metastatic femoral bone lesions: results from a randomised trial. Radiotherapy and Oncology. 2003;69(1):21–31. doi: 10.1016/s0167-8140(03)00232-9 14597353

[10] Van der Wal C, Eggermont F, Fiocco M, Kroon H, Ayu O, Slot A, et al. Axial cortical involvement of metastatic lesions to identify impending femoral fractures; a clinical validation study. Radiotherapy and Oncology. 2020;144:59–64. doi: 10.1016/j.radonc.2019.10.007 31733489

[11] Eggermont F, van der Wal G, Westhoff P, Laar A, de Jong M, Rozema T, et al. Patient-specific finite element computer models improve fracture risk assessments in cancer patients with femoral bone metastases compared to clinical guidelines. Bone. 2020;130:115101. doi: 10.1016/j.bone.2019.115101 31655223

[12] Eggermont F, Derikx LC, Verdonschot N, van der Geest ICM, de Jong MAA, Snyers A, et al. Can patient-specific finite element models better predict fractures in metastatic bone disease than experienced clinicians?: Towards computational modelling in daily clinical practice. Bone Joint Res. 2018;7(6):430–9. doi: 10.1302/2046-3758.76.BJR-2017-0325.R2 30034797 PMC6035356

[13] Sternheim A, Giladi O, Gortzak Y, Drexler M, Salai M, Trabelsi N, et al. Pathological fracture risk assessment in patients with femoral metastases using CT-based finite element methods. A retrospective clinical study. Bone. 2018;110:215–20. doi: 10.1016/j.bone.2018.02.011 29475110

[14] Goodheart JR, Cleary RJ, Damron TA, Mann KA. Simulating activities of daily living with finite element analysis improves fracture prediction for patients with metastatic femoral lesions. Journal of Orthopaedic Research. 2015;33(8):1226–34. doi: 10.1002/jor.22887 25761000

[15] Rajapakse CS, Gupta N, Evans M, Alizai H, Shukurova M, Hong AL, et al. Influence of bone lesion location on femoral bone strength assessed by MRI-based finite-element modeling. Bone. 2019;122:209–17. doi: 10.1016/j.bone.2019.03.005 30851438 PMC6486650

[16] Reeves JM, Knowles NK, Athwal GS, Johnson JA. Methods for Post Hoc Quantitative Computed Tomography Bone Density Calibration: Phantom-Only and Regression. J Biomech Eng. 2018;140(9). doi: 10.1115/1.4040122 29801170

[17] Boomsma MF, Slouwerhof I, van Dalen JA, Edens MA, Mueller D, Milles J, et al. Use of internal references for assessing CT density measurements of the pelvis as replacement for use of an external phantom. Skeletal Radiol. 2015;44(11):1597–602. doi: 10.1007/s00256-015-2206-5 26173417

[18] Habashy AH, Yan X, Brown JK, Xiong X, Kaste SC. Estimation of bone mineral density in children from diagnostic CT images: a comparison of methods with and without an internal calibration standard. Bone. 2011;48(5):1087–94. doi: 10.1016/j.bone.2010.12.012 21185418 PMC4780050

[19] Eggermont F, Verdonschot N, van der Linden Y, Tanck E. Calibration with or without phantom for fracture risk prediction in cancer patients with femoral bone metastases using CT-based finite element models. PLoS One. 2019;14(7):e0220564. doi: 10.1371/journal.pone.0220564 31361790 PMC6667162

[20] Lee DC, Hoffmann PF, Kopperdahl DL, Keaveny TM. Phantomless calibration of CT scans for measurement of BMD and bone strength—inter-operator reanalysis precision. Bone. 2017;103:325–33. doi: 10.1016/j.bone.2017.07.029 28778598 PMC5636218

[21] Boomsma MF, Slouwerhof I, van Dalen JA, Edens MA, Mueller D, Milles J, et al. Use of internal references for assessing CT density measurements of the pelvis as replacement for use of an external phantom. Skeletal radiology. 2015;44(11):1597–602. doi: 10.1007/s00256-015-2206-5 26173417

[22] Weaver AA, Beavers KM, Hightower RC, Lynch SK, Miller AN, Stitzel JD. Lumbar bone mineral density phantomless computed tomography measurements and correlation with age and fracture incidence. Traffic injury prevention. 2015;16(sup2):S153–S60. doi: 10.1080/15389588.2015.1054029 26436225 PMC4602406

[23] Mueller DK, Kutscherenko A, Bartel H, Vlassenbroek A, Ourednicek P, Erckenbrecht J. Phantom-less QCT BMD system as screening tool for osteoporosis without additional radiation. European journal of radiology. 2011;79(3):375–81. doi: 10.1016/j.ejrad.2010.02.008 20223609

[24] Boden SD, Goodenough DJ, Stockham CD, Jacobs E, Dina T, Allman RM. Precise measurement of vertebral bone density using computed tomography without the use of an external reference phantom. Journal of digital imaging. 1989;2(1):31–8. doi: 10.1007/BF03168013 2488150

[25] Weaver AA, Beavers KM, Hightower RC, Lynch SK, Miller AN, Stitzel JD. Lumbar Bone Mineral Density Phantomless Computed Tomography Measurements and Correlation with Age and Fracture Incidence. Traffic Inj Prev. 2015;16 Suppl 2:S153–60. doi: 10.1080/15389588.2015.1054029 26436225 PMC4602406

[26] Winsor C, Li X, Qasim M, Henak C, Pickhardt P, Ploeg H, et al. Evaluation of patient tissue selection methods for deriving equivalent density calibration for femoral bone quantitative CT analyses. Bone. 2021;143:115759. doi: 10.1016/j.bone.2020.115759 33212317

[27] Lee YH, Kim JJ, Jang IG. Patient-specific phantomless estimation of bone mineral density and its effects on finite element analysis results: a feasibility study. Computational and mathematical methods in medicine. 2019;2019. doi: 10.1155/2019/4102410 30719069 PMC6335860

[28] Free J, Eggermont F, Derikx LC, van Leeuwen R, van der Linden YM, Jansen W, et al. The effect of different CT scanners, scan parameters and scanning setup on Hounsfield units and calibrated bone density: a phantom study. Biomedical Physics & Engineering Express. 2018;4(5):055013.

[29] Eggermont F, Derikx LC, Free J, van Leeuwen R, van der Linden YM, Verdonschot N, et al. Effect of different CT scanners and settings on femoral failure loads calculated by finite element models. J Orthop Res. 2018. doi: 10.1002/jor.23890 29508905 PMC6120464

[30] Carpenter RD, Saeed I, Bonaretti S, Schreck C, Keyak JH, Streeper T, et al. Inter-scanner differences in in vivo QCT measurements of the density and strength of the proximal femur remain after correction with anthropomorphic standardization phantoms. Med Eng Phys. 2014;36(10):1225–32. doi: 10.1016/j.medengphy.2014.06.010 25001172 PMC4589175

[31] Giambini H, Dragomir-Daescu D, Huddleston PM, Camp JJ, An KN, Nassr A. The Effect of Quantitative Computed Tomography Acquisition Protocols on Bone Mineral Density Estimation. J Biomech Eng. 2015;137(11):114502. doi: 10.1115/1.4031572 26355694 PMC4844109

[32] Knowles NK, Reeves JM, Ferreira LM. Quantitative Computed Tomography (QCT) derived Bone Mineral Density (BMD) in finite element studies: a review of the literature. J Exp Orthop. 2016;3(1):36. doi: 10.1186/s40634-016-0072-2 27943224 PMC5234499

[33] Alshipli M, Kabir N. Effect of slice thickness on image noise and diagnostic content of single-source-dual energy computed tomography. Journal of Physics: Conf Series. 2017;851(1):012005.

[34] Davis AT, Palmer AL, Pani S, Nisbet A. Assessment of the variation in CT scanner performance (image quality and Hounsfield units) with scan parameters, for image optimisation in radiotherapy treatment planning. Phys Med. 2018;45:59–64. doi: 10.1016/j.ejmp.2017.11.036 29472091

[35] Kalra MK, Maher MM, Toth TL, Schmidt B, Westerman BL, Morgan HT, et al. Techniques and applications of automatic tube current modulation for CT. Radiology. 2004;233(3):649–57. doi: 10.1148/radiol.2333031150 15498896

[36] Birnbaum BA, Hindman N, Lee J, Babb JS. Multi-detector row CT attenuation measurements: assessment of intra- and interscanner variability with an anthropomorphic body CT phantom. Radiology. 2007;242(1):109–19. doi: 10.1148/radiol.2421052066 17185663

[37] Beeres M, Wichmann JL, Paul J, Mbalisike E, Elsabaie M, Vogl TJ, et al. CT chest and gantry rotation time: does the rotation time influence image quality? Acta Radiol. 2015;56(8):950–4. doi: 10.1177/0284185114544242 25140057

[38] Klink T, Regier M, van Stevendaal U, Grass M, Adam G, Begemann P. Accelerating image acquisition in 64-MDCT: the influence of scan parameters on image resolution and quality in a phantom study. Clin Imaging. 2012;36(4):334–44. doi: 10.1016/j.clinimag.2011.11.006 22726972

[39] Sookpeng S, Martin CJ, Gentle DJ. Investigation of the influence of image reconstruction filter and scan parameters on operation of automatic tube current modulation systems for different CT scanners. Radiat Prot Dosimetry. 2015;163(4):521–30. doi: 10.1093/rpd/ncu236 25107439

[40] Wet op de lijkbezorging. 1991.

[41] Derikx LC, Verdonschot N, Tanck E. Towards clinical application of biomechanical tools for the prediction of fracture risk in metastatic bone disease. Journal of biomechanics. 2015;48(5):761–6. doi: 10.1016/j.jbiomech.2014.12.017 25560270

[42] Derikx LC, van Aken JB, Janssen D, Snyers A, van der Linden YM, Verdonschot N, et al. The assessment of the risk of fracture in femora with metastatic lesions: comparing case-specific finite element analyses with predictions by clinical experts. The Journal of bone and joint surgery British volume. 2012;94(8):1135–42. doi: 10.1302/0301-620X.94B8.28449 22844058

[43] Derikx LC, Vis R, Meinders T, Verdonschot N, Tanck E. Implementation of asymmetric yielding in case-specific finite element models improves the prediction of femoral fractures. Computer methods in biomechanics and biomedical engineering. 2011;14(02):183–93. doi: 10.1080/10255842.2010.542463 21337224

[44] Free J, Wang F, Williams N, Gundara JS, Staerkle RF, Hugh TJ, et al. Gallbladder mucosal lesions associated with high biliary amylase irrespective of pancreaticobiliary maljunction. ANZ J Surg. 2018;88(6):E517–E21. doi: 10.1111/ans.14136 28782883

[45] Klein S, Staring M, Murphy K, Viergever MA, Pluim JP. elastix: a toolbox for intensity-based medical image registration. IEEE Trans Med Imaging. 2010;29(1):196–205. doi: 10.1109/TMI.2009.2035616 19923044

[46] Shamonin DP, Bron EE, Lelieveldt BP, Smits M, Klein S, Staring M, et al. Fast parallel image registration on CPU and GPU for diagnostic classification of Alzheimer’s disease. Front Neuroinform. 2013;7:50. doi: 10.3389/fninf.2013.00050 24474917 PMC3893567

[47] Keyak JH, Kaneko TS, Tehranzadeh J, Skinner HB. Predicting proximal femoral strength using structural engineering models. Clin Orthop Relat Res. 2005(437):219–28. doi: 10.1097/01.blo.0000164400.37905.22 16056052

[48] Das IJ, Cheng CW, Cao M, Johnstone PA. Computed tomography imaging parameters for inhomogeneity correction in radiation treatment planning. J Med Phys. 2016;41(1):3–11. doi: 10.4103/0971-6203.177277 27051164 PMC4795414

[49] Schneider W, Bortfeld T, Schlegel W. Correlation between CT numbers and tissue parameters needed for Monte Carlo simulations of clinical dose distributions. Phys Med Biol. 2000;45(2):459–78. doi: 10.1088/0031-9155/45/2/314 10701515

[50] Eggermont PGW F., van der Geest I.C.M., Verdonschot N., Ligthert S., van der Linden Y.M., Tanck E. Implementation of the BOne Strength score for patients with bone metastases in the femur: first results and experiences. NED TIJDSCHR ONCOL. 2021;18:4–11.

[51] Pech P, Bergstrom K, Rauschning W, Haughton VM. Attenuation values, volume changes and artifacts in tissue due to freezing. Acta Radiol. 1987;28(6):779–82. 2962619

[52] Hemmingsson A, Johansson A, Rauschning W. Attenuation in human muscle and fat tissue in vivo and in vitro. Acta Radiol Diagn (Stockh). 1982;23(2):149–51. doi: 10.1177/028418518202300211 7090852

