# Supplementary figures and images for: The effect of variations in CT scan protocol on femoral finite element failure load assessment using phantomless calibration
Source: PLoS One. 2022 Mar 18;17(3):e0265524. doi: 10.1371/journal.pone.0265524 (PMC8932617; doi:10.1371/journal.pone.0265524)

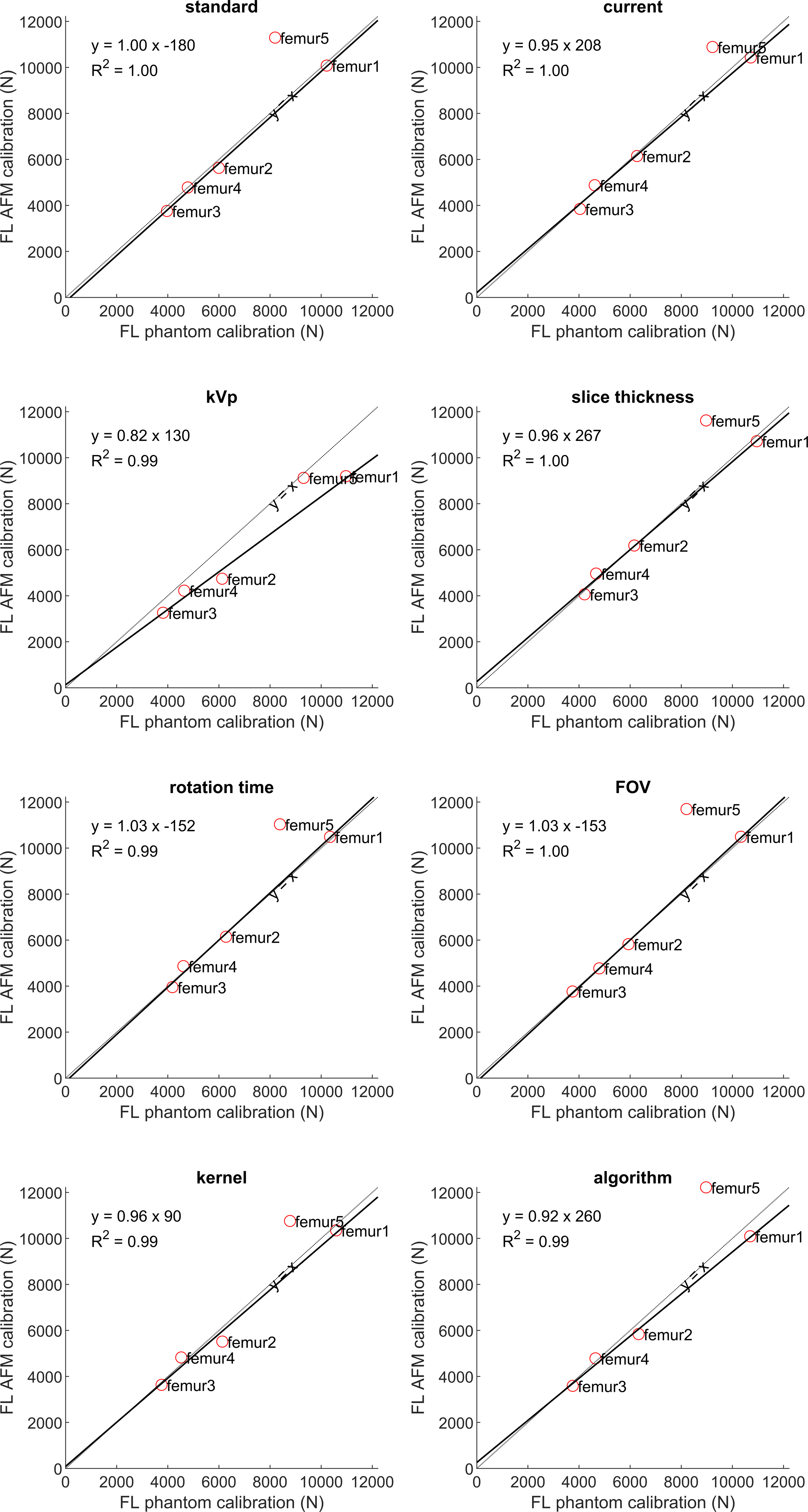

Supplement: S1 Fig — Solid lines show the best-fit lines for each protocol in which femur 5 was excluded due to the accumulation of fat. (TIF) [file pone.0265524.s001.tif]

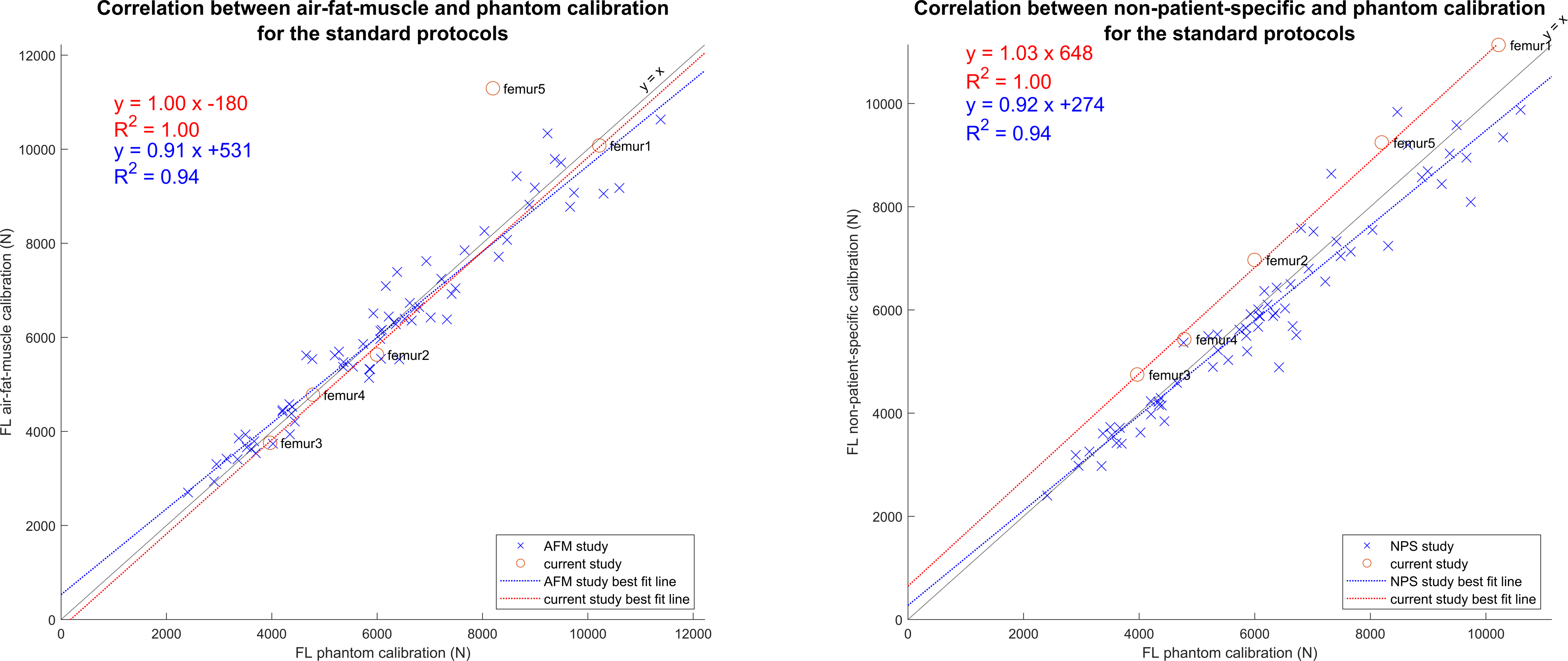

Supplement: S2 Fig — This figure shows that in case of air-fat-muscle calibration four of the five femurs are within the range of the patients studied by Eggermont et al. (red line, n = 4). Only the femur with the deviating fat and muscle balance is deviating. Additionally, it can be seen that the correlations between phantom and air-fat-muscle calibration are comparable when excluding the deviating femur. The femurs calibrated with non-patient-specific calibration seem to deviate more from the femurs of the previous study. This might be explained by the fact that the femurs in the current study are scanned as single legs, whereas the femurs of the previous study were actual patients. When scanning a single leg, the HU will be higher because of less absorption of other tissue (due to a missing contralateral leg). As a result, the failure loads will be too high, for which non-patient-specific calibration apparently cannot correct, whereas air-fat-muscle calibration seems to be able to do this better. (TIF) [file pone.0265524.s002.tif]
