# Supplementary material for: The effect of variations in CT scan protocol on femoral finite element failure load assessment using phantomless calibration
Source: PLoS One. 2022 Mar 18;17(3):e0265524. doi: 10.1371/journal.pone.0265524 (PMC8932617; doi:10.1371/journal.pone.0265524)
Supplement: S1 Table — (DOCX) [file pone.0265524.s003.docx]

S1 Table. mean HUs per protocol for each calibration phantom rod.

|  | **Mean HU** | | | |
| --- | --- | --- | --- | --- |
|  | **Calibration phantom rods** | | | |
| **Protocol** | **0 mg/cm^3^** | **50 mg/cm^3^** | **100 mg/cm^3^** | **200 mg/cm^3^** |
| **standard** | 16.51 | 82.146 | 145.552 | 269.184 |
| **current** | 12.078 | 76.94 | 140.13 | 262.936 |
| **kVp** | 12.194 | 71.132 | 128.382 | 239.126 |
| **slice thickness** | 16.238 | 81.856 | 145.652 | 268.768 |
| **rotation time** | 12.346 | 77.034 | 140.104 | 262.74 |
| **FOV** | 15.816 | 81.352 | 144.74 | 268.04 |
| **kernel** | 16.456 | 82.07 | 145.524 | 269.25 |
| **algorithm** | 16.672 | 82.22 | 145.82 | 269.364 |
